# Preparation and Directed Evolution of Anti-Ciprofloxacin ScFv for Immunoassay in Animal-Derived Food

**DOI:** 10.3390/foods10081933

**Published:** 2021-08-20

**Authors:** Fangyu Wang, Ning Li, Yunshang Zhang, Xuefeng Sun, Man Hu, Yali Zhao, Jianming Fan

**Affiliations:** 1 Key Laboratory for Animal Immunology, Henan Academy of Agricultural Sciences, 116#Huayuan Road, Zhengzhou 450002, China; yunshangzh@163.com (Y.Z.); sunxuefeng2021@126.com (X.S.); human131@163.com (M.H.); 2 Department of Food Nutrition and Health, College of Food Science and Technology, Henan Agricultural University, 63#Agricultural Road, Zhengzhou 450000, China; ln8028@163.com (N.L.); zhaoyali9016@163.com (Y.Z.); 3 China College of Public Health, Zhengzhou University, 100#Kexue Avenue, Zhengzhou 450001, China; fan5746067@126.com

**Keywords:** scFv, ciprofloxacin, recognition mechanism, directional mutagenesis, IC-ELISA

## Abstract

An immunized mouse phage display scFv library with a capacity of 3.34 × 10^9^ CFU/mL was constructed and used for screening of recombinant anti-ciprofloxacin single-chain antibody for the detection of ciprofloxacin (CIP) in animal-derived food. After four rounds of bio-panning, 25 positives were isolated and identified successfully. The highest positive scFv-22 was expressed in *E. coli* BL21. Then, its recognition mechanisms were studied using the molecular docking method. The result showed the amino acid residue Val160 was the key residue for the binding of scFv to CIP. Based on the results of virtual mutation, the scFv antibody was evolved by directional mutagenesis of contact amino acid residue Val160 to Ser. After the expression and purification, an indirect competitive enzyme-linked immunosorbent assay (IC-ELISA) based on the parental and mutant scFv was established for CIP, respectively. The IC50 value of the assay established with the ScFv mutant was 1.58 ng/mL, while the parental scFv was 26.23 ng/mL; this result showed highly increased affinity, with up to 16.6-fold improved sensitivity. The mean recovery for CIP ranged from 73.80% to 123.35%, with 10.46% relative standard deviation between the intra-assay and the inter-assay. The RSD values ranged between 1.49% and 9.81%. The results indicate that we obtained a highly sensitive anti-CIP scFv by the phage library construction and directional evolution, and the scFv-based IC-ELISA is suitable for the detection of CIP residue in animal-derived edible tissues.

## 1. Introduction

Ciprofloxacin (CIP) is a synthetic third-generation fluoroquinolone (FQ) antibiotic that has been developed and is widely used to treat bacterial infections in humans and animals. This antibiotic exerts effects by inhibiting DNA gyrase or topoisomerase II in susceptible bacteria and exhibits high activity against a broad spectrum of Gram-negative and Gram-positive bacteria [1]. However, the unreasonable and extensive use of antibiotics has resulted in the potential for residual antibiotics in food of animal origin, which can damage multiple systems in the body [2,3] and cause bacterial resistance [4,5]. Therefore, the European Union, the Joint FAO/WHO Expert Committee on Food Additives (JECFA, Rome, Italy) and China established maximum residue limits of CIP in animal-derived food to prevent the accumulation of antimicrobial residues, e.g., 100 µg/kg in milk and meat.

By now, many physicochemical methods have been reported for the detection of residues of FQs in foods of animal origin. These analytical methods are highly sensitive and dependable; however, such methods require specialized instrumentation, trained personals, and are time consuming. They are unsuitable for the rapid evaluation of large numbers of samples. Immunoassays, especially the indirect competitive enzyme-linked immunosorbent assay (IC-ELISA), which is based on the principle that antibodies specifically bind to antigens, are considered the most reliable method for detecting antibodies [6,7]. In previous studies, researchers have developed IC-ELISA based on monoclonal antibodies (MAbs) to determine fluoroquinolone in food of animal origin [8,9,10]. Although ELISA is a mature and widely used method, it has many rigorous programs for preparing traditional antibodies (PAbs and MAbs) from antigen-immunized animals [11]. Hence, a simple, rapid, and effective technology for preparing novel antibodies must be developed.

The development of gene engineering techniques facilitated the production of various gene recombinant antibodies, and single-chain variable fragment (scFv) is the most popular format of recombinant antibody that has been successfully constructed by assembling the variable-heavy (VH) region and light chain (VL) domain of an antibody with a flexible linker [12]. The intrinsic properties of scFv antibodies can be improved by various mutagenesis techniques [13]. The recognition property of an scFv antibody can be evolved in vitro [14]. For the evolution of the scFv antibody, its recognition mechanism should be studied first, and binding sites, contact amino acids, and intermolecular forces should be determined [15]. In recent years, molecular docking has been used in analyzing the interactions between ligands and scFv antibodies, and random mutagenesis and site-directed mutagenesis have been used in obtaining scFv mutants [16,17].

Phage display technology (PDT) is the integration of foreign genes into specific coat protein genes of phage and fusion, with coat protein to promote ligand recognition and binding [18,19]. It is considered to be the most suitable technology for the production of single-chain antibodies. The phage antibody library uses genetic engineering methods to amplify VH and VL genes. After random combination, it is inserted into the phage coat protein gene and fused and expressed on the surface of the phage [20]. Specific single-chain antibodies are obtained through specific panning, which is extensively used for preparing antigen-specific artificial antibodies in biomedicine, environmental pollutants analysis, and food safety detection fields. For example, Xu et al. [21] and Zhao et al. [22] obtained the broad-specificity domain antibodies for Bt Cry toxins and pyrethroid pesticides by rounds of specific phage library biopanning, respectively, which are all based on phage antibody library technology.

In this study, an immunized mouse phage display scFv library for screening of anti-CIP phage scFv particles was constructed. Then, we transfected the phage to *E. coli* BL21 for expression, to obtain a highly sensitive anti-CIP scFv. The scFv recognition mechanism was studied through molecular docking, and the sensitivity and cross-reactivity were improved through targeted mutagenesis. Then, IC-ELISA was developed based on the scFv mutant to detect the CIP in animal-derived edible tissues.

## 2. Material and Methods

### 2.1. Reagents and Chemicals

Ciprofloxacin (CIP), enrofloxacin (ENR), sarafloxacin (SAR), difloxacin (DIF), lomefloxacin (LOM), enrofloxacin (ENO), norfloxacin (NOR), amifloxacin (AMI), marbofloxacin (MAR), danofloxacin (DAN), fleroxacin (FLE), ofloxacin (OFL) and pefloxacin (PEF) were obtained from the China Institute of Veterinary Drug Control (Beijing, China). All chemicals and reagents used in this study were at least analytical grade or better. The standard stock solutions of these FQs were prepared with methanol (10 µg/mL), and their working solutions with series concentrations (0.1–200 ng/mL) were diluted from the stock solutions with PBS. All the standard solutions were stored at 4 °C to remain stable for 8 weeks. N-hydroxy succinimide (NHS), γ-aminobutyric acid (4AS), bovine serum albumin (BSA), ovalbumin (OVA), 1-ethyl-3- (3-dimethylaminopropyl)-carbodiimide (EDC), goat anti-mouse IgG horseradish peroxidase conjugate (HRP-IgG), Freund’s complete adjuvant (FCA) and Freund’s incomplete adjuvant (FIA) were from Sigma (St. Louis, MO, USA). PBS (pH 7.2) was prepared by dissolving 0.2 g of KH_2_PO_4_, 0.2 g of KCl, 1.15 g of Na_2_HPO_4_, and 8.0 g of NaCl in 1000 mL of deionized water. Washing buffer (PBST) was PBS buffer containing 0.05% Tween. Coating buffer was 5% MPBS (5% Skim milk powder in PBS). Substrate buffer was 0.1 mol/L citrate (pH 5.5). The substrate system was prepared by adding 200 µL of 1% (*w*/*v*) TMB in DMSO and 64 µL of 0.75% (*w*/*v*) H_2_O_2_ into 20 mL of substrate buffer.

All the restriction enzymes and DNA modification enzymes were molecular biology grade. The RNase prep pure Cell/Bacteria Kit was from Tiangen Biotech Co. Ltd. (Beijing, China). The Prime script RT-PCR Kit, IPTG (isopropyi-β-D-thirgalactopyranoside), X-Gal, pCANTAB5E Vector Cloning kit, horseradish peroxidase-labeled goat anti-GST-tag antibody, restriction enzymes (Sfi I and Not I) and T4 DNA Ligase were from Takara Company (Dalian, China). The EasyPure Quick Gel Extraction Kit, Easy Pure Plasmid Miniprep Kit, express vector PET-32a competent cell BL21(DE3), Fast MultiSite Mutagenesis System and Luria–Bertani culture medium (liquid and solid) were from TransGen Biotech (Beijing, China). The DNA Purification Kit and SDS-PAGE gel preparation kit were from Beijing ComWin Biotech Co. Ltd. (Beijing, China). The synthesis of primers and the analysis of gene sequence were performed at Sangon Biotechnology Co. Ltd. (Shanghai, China).

### 2.2. Synthesis of Antigen

The immunogens CIP-BSA and coating antigens CIP-OVA were synthesized in this study. The details are described below. A mixture of CIP (30 mg), NHS (25 mg) and EDC (30 mg) in 1.5 mL of N, N-dimethylformamide (DMF) was stirred at room temperature overnight. Then, the activated CIP was centrifuged for 15 min (5000 rpm), and the supernatant was added dropwise to 70 mg of BSA dissolved in a solution consisting of 10 mL of PBS and 1 mL of DMF under stirring. The conjugation mixture was stirred at 4 °C for 5 h, and then centrifuged for 10 min (5000 rpm). The supernatant was dialyzed against 0.01 mol/L PBS for 72 h. The dialysis solution was stored at −20 °C. The coating antigens CIP-OVA were prepared as described in the CIP-BSA synthesis section, except that BSA was replaced by OVA.

### 2.3. Immunization

All animal experiments in this study adhered to the Zhengzhou University animal experiment center guidelines and were approved by the Animal Ethics Committee. Six Balb/c female mice (8 weeks old) were induced to express anti-CIP MAbs by immunizing the mice with five rounds of subcutaneous injection of CIP-BSA conjugates. In the first round of immunization, 250 µg of CIP-BSA with FCA was emulsified for subcutaneous multipoint injection, then four subsequent injections were given at 14-day intervals that were emulsified in FCA. Antisera were collected 7 days after the third and fourth immunization, and the antibody titer was determined through indirect ELISA. A week after the fourth round of immunization, booster immunization with 150 µg of CIP-BSA was performed. After 5 days, blood and spleen samples were collected for the construction of the phage display scFv library.

### 2.4. Phage Display scFv Library Construction

Total RNA was extracted from mouse tissues with TRIzol reagent according to the manufacturer’s instructions. Then, total RNA was used as a template in the reverse transcription of cDNA. The sequences of the primers were used in the amplification of the cDNAs of VH and VL genes for scFv construction. The primers used for the amplification of scFv coding sequences were designed according to Table 1 and then spliced to a whole scFv gene through splicing overlap extension PCR (SOE-PCR). The system conditions were as follows: 94 °C for 5 min, 30 cycles at 94 °C for 45 s, 58 °C for 60 s, and 72 °C for 45 s, and final extension at 72 °C for 10 min. Gene fragments encoding VH and VL were amplified and spliced to a single gene by using a DNA linker encoding a pentadeca peptide (Gly4Ser) 3 through primary PCR. The system conditions were 94 °C for 5 min, 30 cycles of at 94 °C for 45 s, 60 °C for 60 s, 72 °C for 45 s, and final extension at 72 °C for 10 min. PCR products were verified through agarose gel electrophoresis, and the relevant fragments were sequenced. The gene fragments were then digested with Sfi I and Not I restriction endonuclease and ligated into pCANTAB5E phagemid vectors. The recombinant vectors were then transformed into *E. coli* TG1 cells. Serial dilutions of 10−1−10−8 were plated onto SOB plates (2% tryptone, 0.5% yeast extract, 0.05% NaCl, 2.5 mM KCl, 10 mM MgCl_2_, and 1.5% Agar powder) that contained 100 µg/mL Amp and 2% Glu. After inoculation, all the plates were incubated overnight in a previously set incubator at 30 °C, then clones were randomly selected and screened for inserts by performing another round of PCR. Finally, the colonies were scraped into 20 mL of 2YT (1.6% Tryptone, 1% yeast Extract, and 0.5% NaCl), named the original antibody library, and stored at −80 °C in 20% glycerol.

### 2.5. Phage scFv Particle Enrichment and Screening

The phage library underwent four rounds of biopanning with coat antigen CIP-BSA for phage scFv particle enrichment. A sterile cell flask was coated with 2 mL of CIP-OVA (the first round was 50 µg/mL, and the remaining three rounds were 25, 12, and 6 μg/mL) in PBS solution at 4 °C and left to stand overnight. The flask was washed five times with PBST solution and blocked with MPBS at 37 °C for 2 h. After being washed with PBST solution, 1 mL of library phage particles was added into a flask for shaking for 1 h at 150 rpm at room temperature, then left to stand for 1 h. The CIP-OVA-bound phage scFv particles were washed with PBST solution and eluted with 1 mL of trypsin solution (1 mg/mL in PBS). The eluent was the first round of enrichment library, and the phage scFv particles were amplified for the next round of enrichment. Four rounds of biopanning were performed. The fourth round of enriched anti-CIP phage particles was infected with *E. coli* TG1 and spread on a TYE-AG medium (contains 100 µg/mL Amp and 1% Glu) for culturing overnight at 37 °C. Individual colonies were randomly picked and grown in 2 × TY-AG medium glucose with 100 µg/mL ampicillin for 16 h at 37 °C and 200 rpm. The next day, 10 µL of culture per well was transferred into another 96-well plate for culturing for 2 h at 37 °C and 200 rpm, and M13KO7 helper phages were added to rescue for 2 h at 37 °C and 200 rpm. The plate was centrifuged at 3300 rpm for 20 min at 37 °C, and the pellets were resuspended with 250 µL/well of 2 × TY-AK medium and cultured overnight at 30 °C and 200 rpm. Finally, the plate was centrifuged at 4 °C and 3300 rpm for 30 min, then the supernatant was used in the monoclonal phage ELISA for CIP.

### 2.6. Colony PCR and Sequencing

The positive phage scFv colonies were cultured in a 2 × TY-AG medium until the logarithmic phase for colony PCR, and the PCR products were examined by 1% agarose gel electrophoresis. The selected positive monoclonal phages were sequenced by Sangon Biotechnology (Shanghai, China) Co., Ltd.

### 2.7. Expression and Purification of scFv

The target gene and prokaryotic expression vector pET-32a were digested with NcolI and NotI restriction enzymes and linked using T4 DNA ligase. Then, the positive recombinant plasmid was used in producing *E. coli* strain BL21 (DE3). The mixture was heat shocked for 90 s at 42 °C, and cultured in a Luria−Bertani (LB) medium (1% tryptone, 0.5% yeast extract, and 1% NaCl) containing 100 µg/mL kanamycin at 37 °C overnight. After the OD_600_ of the bacterium solution reached 0.6–0.8, 1 mmol/L IPTG was added to the culture to induce the expression of scFv. The culture was further grown at 37 °C for 16 h. The supernatant was collected and concentrated 100-fold by using MWCO: 8000–14,000 Da of dialysis bag in PEG/NaCl. The collected pellets were resuspended with PBS for the production of a periplasmic lysate and lysed through sonication for the production of the whole-cell lysate. The supernatant and periplasmic and whole-cell lysates were used in analyzing the solubility of the proteins through SDS-PAGE. Finally, BioMag-SA GST-tag Protein Purification magnetic beads were used to purify the anti-CIP scFv protein.

### 2.8. Denaturation and Renaturation of the scFv Protein

The inclusion bodies were washed five times with PBS containing 0.1% TritonX-100 and 2 mol/mL urea at 2 h intervals; then, the inclusion bodies were solubilized in 20 mL of 8 mol/mL urea solutions and slowly stirred at 4 °C for 16 h. The solubilized solution was centrifuged for 20 min (12,000 rpm). Finally, the solution containing denatured scFv was dialyzed in PBS at 4 °C for 48 h for the removal of urea from the protein solutions.

### 2.9. Characterization of scFv Antibody

Western blot. A volume of scFv solution was added to a nitrocellulose membrane immersed in blocking buffer (4% BSA in PBS (*w*/*v*)) for 1 h. Then, a volume of horseradish peroxidase-labeled anti-GST-tag antibody (1:2000) was added to the block point, and the membrane was incubated for 2 h at room temperature. Finally, a volume of substrate solution (4-chloro-1-naphthol) was added for the visualization of the result.

Indirect competitive ELISA. The purified anti-CIP scFv was used in establishing IC-ELISA. Briefly, 100 µL/well of CIP-OVA solution was coated into 96-well plates overnight at 4 °C; then, the plates were washed with PBST solution and blocked with 300 µL per well of 5% MPBS at 37 °C for 1 h. scFv (100 µL/well) previously diluted with PBS and a series of CIP standard concentrations (200, 100, 80, 50, 20, 10, 5, 2, 1, and 0.1 ng/mL) were washed with PBST and then mixed. The plates were incubated at 37 °C for 1 h, then washed with PBST. Avidin conjugated with horseradish peroxidase (100 µL/well; 1/2000 dilution in PBS) was added to the wells and incubated at 37 °C for 30 min. The wells were then washed five times with PBST, and 100 µL/well of TMB substrate was added and incubated for 10 min in the dark at room temperature. Color reaction was stopped with the addition of sulfuric acid (2 mol/L, 50 µL/well). Finally, absorbance was measured at 450 nm with an automatic microplate reader (Thermo, Waltham, MA, USA). The IC50 value, assay dynamic range, and limit of detection (LOD) served as the criteria for evaluating IC-ELISA. The inhibition ratios of anti-CIP scFv, IC10, IC20, IC50, and IC80 were calculated using the formula [(P-S-N)]/(P-N)] × 100%, where P is the OD_450_ value of the positive sample (50 µL of anti-CIP scFv mixed with 50 µL of CBS), S is the OD_450_ value of the standard (50 µL of scFv mixed with 50 µL of the serial concentration of CIP), and N is the OD_450_ value of the negative control (100 µL of CBS).

### 2.10. Homology Modeling and Molecular Docking

In this experiment, the possible template sequences of the anti-CIP scFv model were searched in the NCBI database (https://www.ncbi.nlm.nih.gov/ (accessed on 1 February 2021)), and sequence comparison was performed in the BLAST section for a selection template of a high consistency with the anti-CIP scFv model. The anti-CIP scFv template sequence is as follows: (sense): 5′-TCAAGTGTAAGTTACATGCCATGGTACCAGCAG-3′ and (antisense): 5′-TCTTGGCTTCTGCTGGTACCATGGCATGTAACTTACACT-3′. The sequence with a high score and low e-value was used as a template sequence for anti-CIP scFv model building. Then, the SWISS MODEL online server was used in the homology modeling of anti-CIP scFv. To verify the reliability of the homology modeling results and determine the best model structure, we used Procheck, Verify3D, and ERRAT programs in the evaluation of the consistency of the constructed an anti-CIP scFv homology model and selected the best receptor model for further molecular docking study. To study the binding mode of CIP with anti-CIP-scFv and find key residues, we used MOE 2015.10 in exploring the molecular docking of CIP with scFv. In the Dock module, CIP was docked into the active site of anti-CIP-scFv through the method of Induced Fit and under Amber10: EHT forcefield. The docking ligand, which had 30 docking conformations after default parameters were used, were used for further analysis.

### 2.11. Directional Mutagenesis of scFv Antibody

The binding affinity of CIP with scFv-CIP antibody was improved through the virtual mutation of the potential key residues of scFv-CIP. The process was based on the study of the binding mode of anti CIP scFv with CIP. MOE 2015.10 software was used in conducting the virtual mutation of amino acid residues that affect the binding of CIP with scFv and directly replace them with other amino acids. In this study, Ser was used to replace Val160 for the production of the mutant of scFv-CIP antibody. Then, the structure of the virtual mutation scFv model was optimized, and a stable scFv mutation model was obtained. Subsequently, the docking study of CIP with scFv mutation was performed through the method of Induced Fit, and 30 conformations were obtained using the default parameters. During the experiments, the scFv gene in the express vector scFv-pCANTAB5E was mutated directly for the production of a mutated express vector with a fast-multisite mutagenesis system according to the manufacturer’s recommended protocol. Then, the mutated express vector was expressed for the production of scFv mutant using the procedures described above. The scFv mutant was identified and analyzed through SDS-Page and IC-ELISA [6].

### 2.12. Sample Preparation and Cross-Reactivity Analysis

Beef, pork, milk, and chicken samples were obtained from a local market. CIP (1000 μg/mL, prepared in PBS) was added to each sample for the production of spiked concentrations of 0, 50, 100, and 200 μg/kg. Aliquots of the homogenized tissue samples (1 g of wet mass) were transferred to a 50 mL centrifuge tube. Exactly 5 mL of 5% trichloroacetic acid and 10 mL of 0.2 M PBS were mixed with the tissue sample, and the mixture was incubated for 1 h at 60 °C. Subsequently, the suspension was centrifuged at 5000× *g* for 20 min. The supernatant was separated and diluted tenfold with deionized water. The aliquots (100 mL each) were distributed into the microtiter plate. The CIP standards of different concentrations (0, 50, 100, and 200 μg/L) were added to milk samples, which were then defatted by centrifugation at 5000× *g* for 20 min at 4 °C. After 60 µL of sodium nitroprusside (0.36 mol/L) and 60 μL of zinc sulfate (1.04 mol/L) were added to 2 mL of each defatted milk sample, the samples were vortexed for 1 min and then centrifuged at 5000× *g* for 20 min at 4 °C. The supernatant was removed and diluted tenfold with PBS for analysis. Recoveries were calculated on the basis of the standard curve constructed by IC-ELISA.

The specificity of the scFv under optimized conditions was evaluated by measuring cross-reactivity (CR) with a group of structurally related compounds, including 12 other analogs such as enrofloxacin, danofloxacin, and fleroxacin. The CRs of anti-CIP scFv for CIP analogues were calculated using the formula: [CR (%) = IC50 (CIP)/IC50 (CIP analogue)] × 100%.

### 2.13. Statistical Analysis

The statistical software SPSS 16.0 and data processing system 14.0 (DPS) were used for statistics. Values are expressed as mean ± standard deviation. All data are suitable for analysis without any conversion.

## 3. Results and Discussion

### 3.1. Construction of Phage Display scFv Library

**Figure 1:**
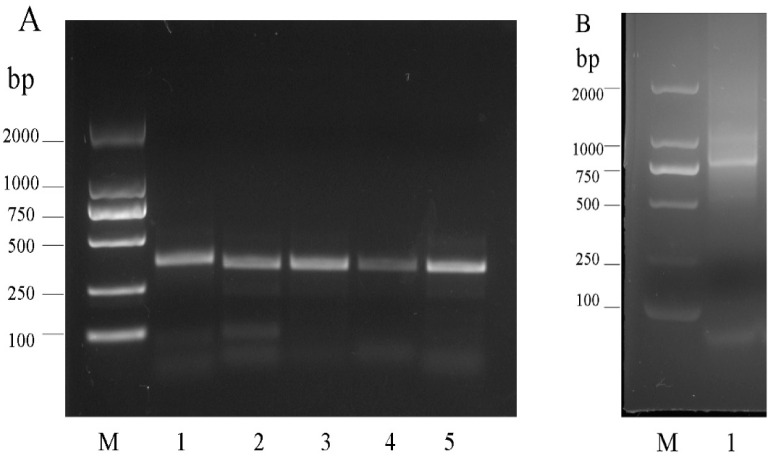
Amplification of heavy and light chains. Agarose gel electrophoresis of the amplified antibody variable fragments. (**A**) PCR amplification of VH and VL. Lane 1: PCR products of VH, about 350 bp. Lane 2. Lane 3 and Lane 4: PCR products of VL, about 330 bp. (**B**) Amplification of scFv by overlap PCR, about 780 bp.

Compared with conventional antibodies (monoclonal antibodies and polyclonal antibodies), scFv can be produced on a large scale in prokaryotic and eukaryotic systems, so it is cheap and saves time [23,24]. In addition, the scFv antibody can be studied at the molecular level (homologous modeling and molecular docking), and its antigen binding affinity can be improved through gene mutation and gene reforming [15]. In this study, a scFv library for mouse phage display was constructed, and total RNA was extracted from the spleen of immunized mice and then reverse-translated to cDNA. The VH and VL coding sequences were amplified using the cDNA as the template, and a complementary linker sequence was added. The amplified and purified NcoI-VL-linker and linker-VH-NotI were spliced to whole scFv genes through SOE-PCR. As shown in Figure 1, the amplified VH, VL, and scFv DNAs were approximately 350, 330, and 780 bp long, respectively. The purified scFv and pCANTAB5E vectors were digested with SfiI and NotI. T4 DNA ligase was used to ligate the products, and recombinant plasmids were translated into *E. coli* TG1 cells, and a library with a capacity of 3.34 × 10^9^ CFU/mL was constructed successfully.

### 3.2. Panning of Phage-Displayed Antibody Libraries

**Figure 2:**
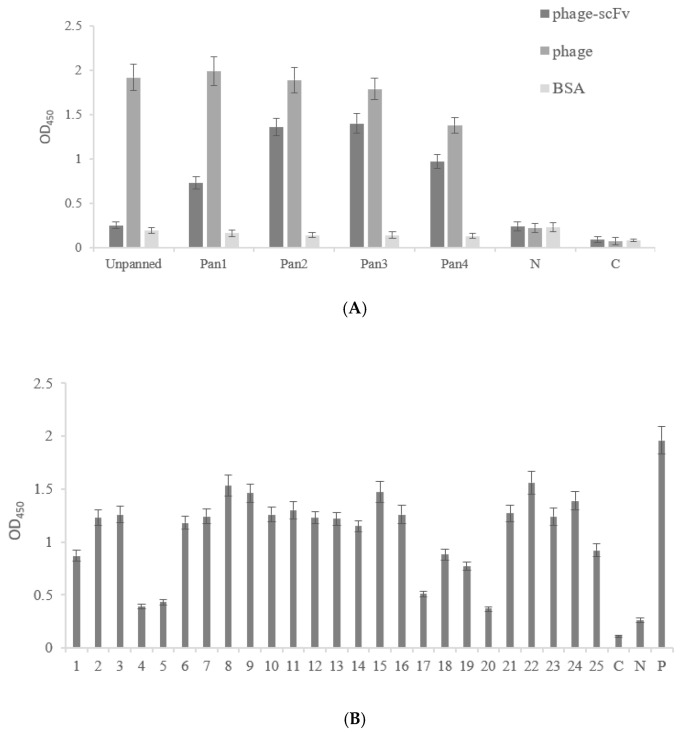
Phage-ELISA. (**A**) The enrichment of specific scFv in each library after four rounds panning. (**B**) Binding activity of scFv antibodies to CIP. 1–25: scFv antibodies from randomly selected clones from the 4th panning; C: blank control; N: negative control, BSA; P: positive control, cell supernatant.

For the production of highly specific antibodies, the washing steps were progressively increased, whereas the concentrations of coated CIP-OVA were decreased (Table 2) as described by Li et al. [6]. It can be seen from Figure 2A that after the first three rounds of panning, the antibody response signal gradually increased, and decreased after the fourth round of panning, indicating that the phage antibody library was effectively enriched with specific phage particles after four rounds of panning. On the plate after the fourth round of panning, 25 phage colonies were randomly selected for Phage-ELISA to analyze the binding ability of ciprofloxacin. The results are shown in Figure 2B. Clone scFv-22, which showed relatively stable and high binding abilities, was selected for further study.

### 3.3. Expression, Purification of the scFv-22

**Figure 3:**
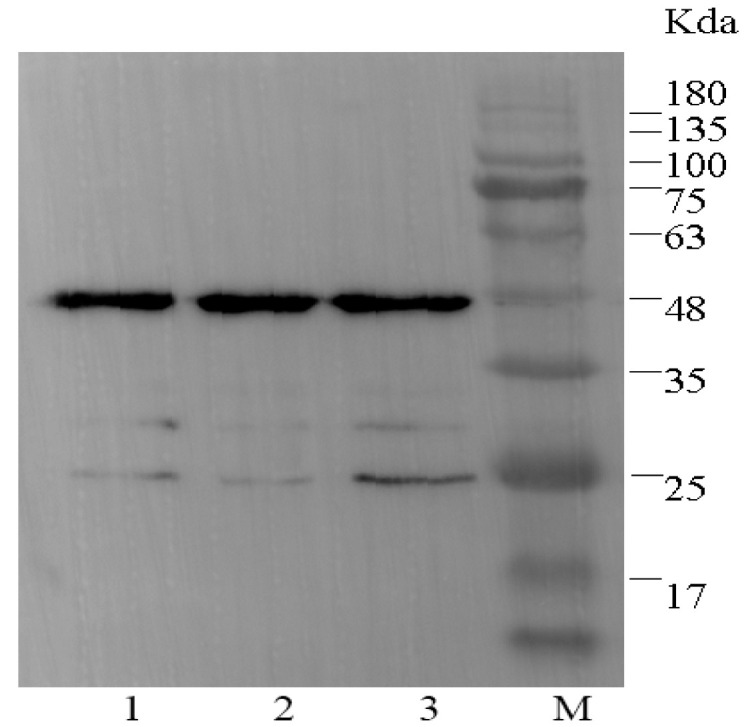
Analysis of scFv-22 by Western blot. Lane 1–3: scFv-22 induced expression. Lane M: Protein 180 marker.

**Figure 4:**
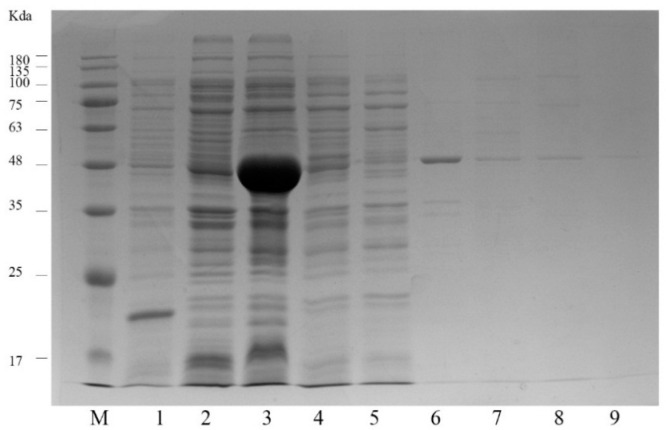
Analysis of scFv by SDS-PAGE. Lane M: Protein 180 marker. Lane 1: PET-32a vector. Lane 2–3: After induction of expression, the supernatant and precipitate obtained by sonication. Lane 4–5: The supernatant obtained after combining with the magnetic beads. Lane 6–9: Supernatant after washing with 20 mM, 100 mM, 200 mM, 250 mM imidazole, respectively.

As is well known, IPTG concentration, post-induction time, and incubation temperature are the main factors for optimizing protein expression [25]. In the preliminary experiments of this study, the highest scFv expression level was obtained at the following conditions: 37 °C, 16 h, and 1 mM IPTG. Under optimal expression conditions, the scFv-22 antibody fragments were expressed in *E. coli* HB2151, and proteins were determined through Western blotting, as shown in Figure 3. The results indicated that the scFv-22 antibody (approximately 47 kDa) was expressed successfully. Anti-CIP scFv proteins were purified using BioMag-SA GST-tag Protein Purification magnetic beads according to the manufacturer’s instructions. The purity of protein solution was confirmed through SDS-PAGE, as shown in Figure 4, and then the purified protein was stored at −2 °C.

### 3.4. IC-ELISA for CIP and Its Analogues Based on scFv-22

**Figure 5:**
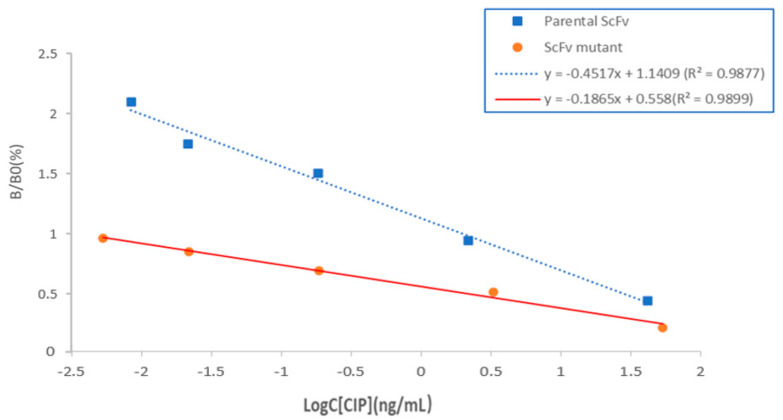
Standard curves of the competitive ELISA for CIP. X-axis shows the logarithm concentration of CIP, Y-axis represents the inhibition rate (B/B0). B0 and B are the absorbance values obtained from binding at zero and certain concentrations of CIP standard.

The performance of the purified scFv-22 was evaluated using IC-ELISA. The optimum concentration of scFv-22 was 0.25 µg/mL, producing an OD_450_ of 1.0 at 4 µg/mL of CIP-OVA coating concentration through checkerboard titration. Under the optimal conditions, the regression curve equation of CIP-scFv was y = −0.4517x + 1.1409 (R^2^ = 0.9877), as shown in Figure 5. The IC50 value of the assay established with scFv-22 was 26.23 ng/mL, demonstrating that scFv can be used in detecting CIP. The linear range of the assay established with scFv ranged from 5.68 ng/mL to 201.55 ng/mL.

### 3.5. Homology Modeling and Molecular Docking

In this experiment, given the results of parameter evaluation, such as sequence identity and structural similarity, the protein PDB ID: 3UZQ was selected as the template sequence, which has the highest sequence identity (78.8%) with the target sequence anti-CIP-scFv. Subsequently, a stable model of single-chain antibody was established using the SWISS MODEL homology modeling software. The single-chain antibody of anti-CIP-scFv was connected by VH and VL through three connecting peptides (Gly4Ser) and had a typical single-chain antibody structure with anti-parallel β-sheet and loop regions.

We evaluated the model to verify its reliability. The Procheck program was used in evaluating the three-dimensional structure of the anti-CIP-scFv model. The Ramachandran plot showed that 91.7% of the amino acids residues in the model were located in the core region, 7.8% in the allowable region, and only 0.5% in the forbidden zone of the twist angle. The results showed that the dihedral angles of 99% of protein residues in scFv model were within the reasonable range and conformed to the rule of stereochemical energy. The result of Verify-3D showed that the average 3D–1D score of 99.58% of amino acid residues in the scFv model was greater than 0.2, and the model passed the Verify-3D test. The Errat result showed that the overall quality factor was 87.82. Therefore, the experimental model of anti-CIP-scFv has high reliability and can be used in the molecular docking of the CIP antigen.

**Figure 6:**
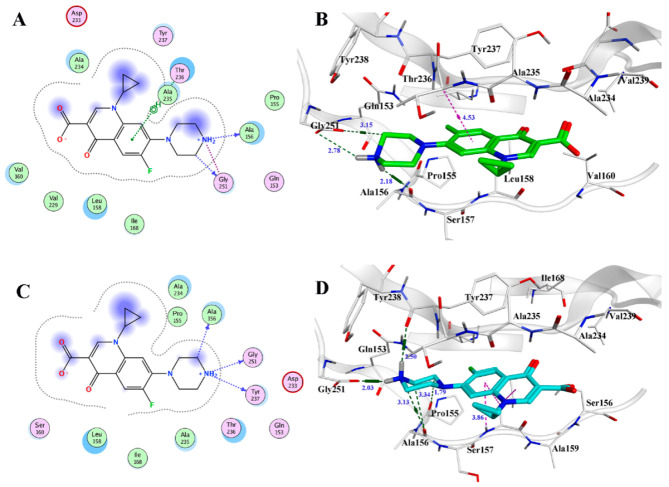
(**A**) The binding mode between parental scFv and CIP, (**B**) the interaction between parental scFv and CIP binding site; (**C**) the binding mode between scFv mutant and CIP, (**D**) the interaction between scFv mutant and CIP binding site.

The docking result of scFv-22 with CIP is shown in Figure 6. The active site of scFv-22 consists of residues Gln153, Pro155, Ala156, Leu158, Val160, Ile168, Val229, Asp233, Ala234, Ala235, Thr236, and Tyr237. The carboxyl group of CIP can form a 2.78 Å and 3.15 Å hydrogen bond with the residue Gly251. The quinoline structure is located in the hydrophobic cavity formed by residues Gln153, Pro155, Ala156, Ile168, Gly251, and Thr236. The cyclopropyl group can interact with the residues Ala235 and forms hydrophobic interaction with Tyr238. Thus, these forces may be the main reason for the increased ability to bind to CIP. However, the hydrophilic carboxyl group in CIP and the carbonyl group on the quinoline are close to the hydrophobic residue Val160. We preliminarily speculated that if the residue Val160 is transformed to a hydrophilic amino acid, it will promote the combination of CIP to scFv. When Val160 was substituted by Ser, the total binding energy decreased from −5.23 to −7.91 kcal/mol, and the number of hydrogen bonds and the amino acids forming hydrophobic interaction all increased in the binding site (Figure 6B,D), indicating that the intermolecular forces of scFv-CIP increased. Therefore, Val160 was substituted with Ser for the directional mutagenesis of the scFv antibody in the present study.

### 3.6. Characterization of Mutant scFv

The performance of purified scFv mutant was evaluated through SDS-PAGE and IC-ELISA. Under the same conditions as the parental scFv, the regression curve equation of the mutant scFv was y = −0.1865x + 0.558 (R^2^ = 0.9899), as shown in Figure 5. The IC50 value of the assay established with the scFv mutant was 1.58 ng/mL, indicating that the affinity of the scFv mutant increased 16.6 times compared with that of the parental scFv. The mutant had a higher affinity and better sensitivity than the original antibody, indicating that the parental scFv antibody was evolved successfully.

### 3.7. Precision and Recovery

Spiking and recovery tests were conducted for the assessment of the feasibility of IC-ELISA. During the tests, the mutant scFv was used in detecting CIP in spiked samples, and no positive results were obtained for the non-spiked samples. All samples spiked with CIP showed good agreement between the spiking level and concentration detected, as shown in Figure 3. In the intra-assay, the mean recovery for CIP ranged from 73.80% to 121.58% and the RSD values ranged between 2.01% and 7.35% (based on triple measurements within a day). In the inter-assay, the mean recovery for CIP ranged from 75.29% to 123.35%, and RSD values ranged between 1.49% and 9.81% (based on triple measurements in 3 days). As demonstrated with the samples spiked with CIP, the IC-ELISA method provided satisfactory results for the detection of CIP residues in milk and food animal tissues. The cross-reactivity of the CIP-scFv with danofloxacin, enrofloxacin, and fleroxacin and many more were tested through IC-ELISA. As shown in Figure 4, the CIP-scFv showed low cross-reactivity with other fluoroquinolones, indicating that scFv is highly specific for CIP.

## 4. Conclusions

A highly sensitive anti-CIP single-chain antibody was obtained through phage display and directional evolution, and a rapid and highly sensitive IC-ELISA method for detecting CIP residues in products of animal origin was developed. The method showed good stability, reproducibility, and accuracy for detecting CIP, indicating a wide application prospect for the rapid and sensitive detection of antibiotic residues in animal-derived food.

## Figures and Tables

**Table 1:** Nucleotide primer sequences.

Primer Names	Nucleotide Sequences (5′→3′)
VH for	GCGGCCCAGCCGGCCATGGCCGARGTGAAGCTGGTGGARTCTGGR
VH back	AGCGGCGGTGGCGGTTCTGGAGGCGGCGGTTCTGAYATGCAGATGACMCAG
VL for	AGCGGCGGTGGCGGTTCTGGAGGCGGCGGTTCTRAMATTGTGMTGACCCAATC AGCGGCGGTGGCGGTTCTGGAGGCGGCGGTTCTGAYATGCAGATGACMCAGWC
VL back	ACTAGTCGCGGCCGCGTCGACAGCMCGTTTBAKYTCTATCTTTGT ACTAGTCGCGGCCGCGTCGACAGCMCGTTTCAGYTCCARYTT
scFv for	CGCAATTCCTTTAGTTGTTCCTTTCTATGCGGCCCAGCCGGCCATGGCC
scFv back	GGTTCCAGCGGATCCGGATACGGCACCGGACTAGTCGCGGCCGCGTCGAC

**Table 2:** The library size and phage titer of each panning.

Rounds	Coated Antigen	Coating Concentrations (µG/WELL)	Input	Output	Output/Input
1	CIP-OVA	50	4 × 10^11^	2.9 × 10^5^	7.55 × 10^−7^
2	CIP-OVA	25	4 × 10^11^	5.6 × 10^6^	1.65 × 10^−5^
3	CIP-OVA	12	4 × 10^11^	2.9 × 10^8^	5.9 × 10^−4^
4	CIP-OVA	6	4 × 10^11^	3.34 × 10^9^	6.15 × 10^−3^

## References

[1] Dalhoff A. (2015). Antiviral, antifungal, and antiparasitic activities of fluoroquinolones optimized for treatment of bacterial infections: A puzzling paradox or a logical consequence of their mode of action?. Eur. J. Clin. Microbiol. Infect. Dis..

[2] Bird S.T., Etminan M. (2013). Risk of acute kidney injury associated with the use of fluoroquinolones. CMAJ.

[3] Patel K., Goldman J.L. (2016). Safety Concerns Surrounding Quinolone Use in Children. J. Clin. Pharmacol..

[4] Li J., Hao H. (2017). The effects of different enrofloxacin dosages on clinical efficacy and resistance development in chickens experimentally infected with Salmonella Typhimurium. Sci. Rep..

[5] Xu L., Wang H. (2013). Integrated pharmacokinetics/pharmacodynamics parameters-based dosing guidelines of enrofloxacin in grass carp Ctenopharyngodon idella to minimize selection of drug resistance. BMC Vet. Res..

[6] Cui L., Jinxin H. (2016). Preparation of a Chicken scFv to Analyze Gentamicin Residue in Animal Derived Food Products. Anal. Chem..

[7] Abdelwahab M., Loa C.C. (2015). Recombinant nucleocapsid protein-based enzyme-linked immunosorbent assay for detection of antibody to turkey coronavirus. J. Virol. Methods.

[8] Huang B., Yin Y. (2010). Preparation of high-affinity rabbit monoclonal antibodies for ciprofloxacin and development of an indirect competitive ELISA for residues in milk. J. Zhejiang Univ. Sci. B.

[9] Fan G.-Y., Yang R.-S. (2012). Development of a class-specific polyclonal antibody-based indirect competitive ELISA for detecting fluoroquinolone residues in milk. J. Zhejiang Univ. Sci. B.

[10] Zhang H.-T., Jiang J.-Q. (2011). Development of an indirect competitive ELISA for simultaneous detection of enrofloxacin and ciprofloxacin. J. Zhejiang Univ. Sci. B.

[11] Li C., Luo X. (2019). A Class-Selective Immunoassay for Sulfonamides Residue Detection in Milk Using a Superior Polyclonal Antibody with Broad Specificity and Highly Uniform Affinity. Molecules.

[12] Makvandi-Nejad S., Sheedy C. (2010). Selection of single chain variable fragment (scFv) antibodies from a hyperimmunized phage display library for the detection of the antibiotic monensin. J. Immunol. Methods.

[13] Norihiro K. (2008). Anti-estradiol-17beta single-chain Fv fragments: Generation, characterization, gene randomization, and optimized phage display. Steroids.

[14] Kobayashi N., Oyama H., Kato Y., Goto J., Söderlind E., Borrebaeck C.A. (2010). Two-step in vitro antibody affinity maturation enables estradiol-17beta assays with more than 10-fold higher sensitivity. Anal. Chem..

[15] Liu J., Zhang H.C. (2016). Production of anti-amoxicillin ScFv antibody and simulation studying its molecular recognition mechanism for penicillins. J. Environ. Sci. Health Part B Pestic. Food Contam. Agric. Wastes.

[16] Wen K., Nolke G. (2012). Improved fluoroquinolone detection in ELISA through engineering of a broad-specific single-chain variable fragment binding simultaneously to 20 fluoroquinolones. Anal. Bioanal. Chem..

[17] Tao X., Chen M. (2013). Chemiluminescence competitive indirect enzyme immunoassay for 20 fluoroquinolone residues in fish and shrimp based on a single-chain variable fragment. Anal. Bioanal. Chem..

[18] Kumar R., Parray H.A. (2019). Phage display antibody libraries: A robust approach for generation of recombinant human monoclonal antibodies. Int. J. Biol. Macromol..

[19] Zhao A., Tohidkia M.R., Siegel D.L., Coukos G., Omidi Y. (2016). Phage antibody display libraries: A powerful antibody discovery platform for immunotherapy. Crit. Rev. Biotechnol..

[20] Arap M.A. (2005). Phage display technology: Applications and innovations. Genet. Mol. Biol..

[21] Xu C., Miao W. (2019). Construction of an immunized rabbit phage display antibody library for screening microcystin-LR high sensitive single-chain antibody. Int. J. Biol. Macromol..

[22] Zhao Y., Liang Y. (2016). Isolation of broad-specificity domain antibody from phage library for development of pyrethroid immunoassay. Anal. Biochem..

[23] Zhang X., Zhang C. (2010). Construction of scFv phage display library with hapten-specific repertories and characterization of anti-ivermectin fragment isolated from the library. Eur. Food Res. Technol..

[24] Chaisri U., Chaicumpa W. (2018). Evolution of Therapeutic Antibodies, Influenza Virus Biology, Influenza, and Influenza Immunotherapy. BioMed Res. Int..

[25] Dong S., Bo Z. (2018). Screening for single-chain variable fragment antibodies against multiple Cry1 toxins from an immunized mouse phage display antibody library. Appl. Microbiol. Biotechnol..

